# Syphilis prevalence and risk factors among young men presenting to the Brazilian Army in 2016: Results from a national survey

**DOI:** 10.1097/MD.0000000000013309

**Published:** 2018-11-21

**Authors:** Leonardo Rapone da Motta, Rosa Dea Sperhacke, Aline de Gregori Adami, Sérgio Kakuta Kato, Andréa Cristina Vanni, Machline Paim Paganella, Maria Cristina Pimenta de Oliveira, Silvana Pereira Giozza, Alessandro Ricardo Caruso da Cunha, Gerson Fernando Mendes Pereira, Adele Schwartz Benzaken

**Affiliations:** a HIV/AIDS Research Laboratory (LPHA), Life Sciences Knowledge Area. University of Caxias do Sul (UCS), Caxias do Sul; b Graduate Program in Rehabilitation Sciences, Federal University of Health Sciences of Porto Alegre, Porto Alegre, RS; c Department of Surveillance, Prevention and Control of STI, HIV/AIDS and Viral Hepatitis, Secretariat of Health Surveillance, Ministry of Health of Brazil, Brasília, DF, Brazil.

**Keywords:** army, conscripts, prevalence, sexual behavior, MSM, syphilis, young men

## Abstract

The Conscripts Survey has been conducted periodically by the Brazilian Department of Sexually Transmitted Infections (STIs), AIDS, and Viral Hepatitis (DIAHV) in collaboration with the Brazilian Ministry of Defense for over 2 decades. It aims to assess the syphilis prevalence and obtain data on knowledge regarding STIs and their risk factors among conscripts enlisted for the Brazilian Army.

This cross-sectional study was conducted among conscripts across Brazil aged 17 to 22 years from August to December 2016. It included a self-reported questionnaire and blood testing for syphilis, HIV, and hepatitis B and C.

In total 38,247 conscripts were enrolled; after exclusion due to a lack of information, 37,282 (93.2%) conscripts were included. The estimated syphilis prevalence rates were: 1.63%, 1.09%, and 0.62% for screened, confirmed, and active syphilis, respectively. Among those with active syphilis, 81.1% reported not having syphilis infection in their lifetime. Higher confirmed syphilis prevalence rates were observed in the South region, followed by North and Southeast regions. Independent factors associated with confirmed syphilis infection were: self-reported STIs in one's lifetime (odds ratio [OR] = 7.24; *P* < .001), same-sex sexual relationships (OR = 3.43; *P* = .001), and having the 1st sexual intercourse encounter before 15 years of age (OR = 2.62; *P* = .04). The proportion of conscripts who reported having sex with other men (MSM) was 4.3%, and the estimated syphilis prevalence in this group was 5.23%, 4.61%, and 3.60% for screened, confirmed, and active syphilis, respectively. The sexual behaviors most frequently associated with confirmed syphilis were: sexual relationship with casual partners in the last year (*P* < .001), same-sex sexual relationships (*P* < .001), more than 10 partners (*P* = .006), and having sexual intercourse before 15 years of age (*P* = .003). Although not significant, only 25.4% of the conscripts who had a confirmed syphilis reported the use of condoms with steady partners, 32.4% with casual partner, and 24.3% with any partner.

We found that syphilis is on the rise among the young Brazilian male population. The increase in its prevalence, particularly among MSM, highlights the need for urgent public health interventions, action plans, and implementation of risk reduction strategies aimed at this population.

## 1 Introduction

Almost a century after the discovery of the drug of choice for syphilis treatment, penicillin, syphilis infection is re-emerging as a public health problem worldwide.^[1,2]^ The World Health Organization (WHO) estimated that 5.6 million new cases of syphilis occurred among adolescents and adults 15 to 49 years of age worldwide in 2012 with a global incidence rate of 1.5 cases per 1000 population.^[3]^

In Brazil, 87,593 new acquired cases of syphilis were diagnosed in 2016. The acquired syphilis detection rate in Brazil has dramatically increased from 2 cases per 100,000 population in 2010 to 42.5 cases per 100,000 population in 2016. This increase can be attributed, at least in part, to an increase in the coverage of syphilis testing, the expansion of the use of rapid tests for syphilis, a reduction in condom use, the lack of awareness regarding sexually transmitted infections, the lack of willingness among health care professionals to administrate penicillin in primary care services, the worldwide shortage of penicillin, and the enhancement of the Brazilian surveillance system.^[4]^

Globally, the syphilis seroprevalence is high especially in key populations (men who have sex with men [MSM], sex workers, people who use drugs, transgender people, and incarcerated individuals).^[5]^ In 2014, 30 countries reported syphilis data for MSM with a median seroprevalence of 5.3% (overall range 0.3–32.2%).^[5]^ Recent studies show that the syphilis epidemic is also largely concentrated among MSM in Brazil.^[6–9]^ Additionally, syphilis infection is of major concern as its ulcerative lesions facilitate HIV acquisition. The risk of acquiring HIV infection through sexual intercourse is increased 3 to 5 times in individuals who are infected with syphilis compared to those who are not.^[10]^

The Conscripts Survey has been conducted periodically by the Brazilian Department of Sexually Transmitted Infections (STI), HIV/AIDS, and Viral Hepatitis (DIAHV) in collaboration with the Ministry of Defense for over a decade. It aims to assess the STI prevalence and obtain data on knowledge regarding STIs and their risk factors among conscripts enlisted for the Brazilian Army.^[11,12]^ Knowledge regarding syphilis was 1st assessed in the 6th edition of the Conscripts Survey performed in 2002, while syphilis seroprevalence was included in the 7th edition in 2007.^[13]^ Data from these surveys are used to monitor syphilis trends in young males and are applied as a proxy to estimate the prevalence of syphilis among adults in the overall population.

Knowledge about the epidemiologic distribution patterns of syphilis infection in Brazilian regions is necessary to understand the dynamics of this disease throughout the country, as well as to develop prevention strategies and public health policies. This study aimed to present the estimated prevalence of syphilis and the risk factors associated with syphilis based on the results of the 8th edition of the Conscript Survey performed in 2016 based on the geographic region in the country.

## 2 Methods

### 2.1 Subject selection and sampling

This cross-sectional study was conducted among young men across Brazil aged 17 to 22 years who were in compulsory enlistment for military service from August to December 2016.

In total, 39,996 conscripts were selected to participate in the survey by following a sampling plan based on stratification in 2 selection stages previously described.^[14]^ Exclusion criteria for this study were: illiterate conscripts; conscripts outside the age range of 17 to 22 years; lack of information regarding age, origin (municipality), and educational levels; and refusal to sign the informed consent.

### 2.2 Data collection and laboratory assays

The study participants completed a self-reported anonymous questionnaire and provided blood samples for HIV, hepatitis B and C, and syphilis infection testing. The questionnaire contained 74 questions and included questions about sociodemographic characteristics, sexual behavior practices, problems related to STIs, and the use of licit/illicit drugs.

Participants were classified as MSM if they reported having sex “only with men” and “with men and women.” All questionnaires were processed at the Laboratório de Pesquisa em HIV/AIDS (Universidade de Caxias do Sul, Caxias do Sul, RS, Brazil) using OpenText TeleForm 11.1 (Waterloo, ON, Canada).

Specimens were obtained from recruited conscripts and tested for syphilis using the treponemal test Architect Syphilis TP (Abbott Laboratories, Wiesbaden, Germany). Samples that were nonreactive on this assay were classified as syphilis negative, and no additional tests were performed on these samples. Specimens with positive results were classified as screened for syphilis and subjected to the nontreponemal V.D.R.L. test (Wiener Lab, Rosario, Argentina). Specimens with positive results on both tests were classified as confirmed syphilis. Specimens with discordant results, between the nontreponemal and treponemal tests, were subjected to the treponemal Imuno-Com FTA-Abs Sífilis (Wama Diagnóstica, São Carlos, SP, Brazil) test. Specimens with positive results on both treponemal assays were classified as confirmed syphilis specimens; otherwise, a negative result was reported. Subjects were considered to have active syphilis if they had a positive Architect Syphilis TP test and a venereal disease research laboratory (VDRL) with a titer ≥1:8. All syphilis tests were performed according to the manufacturer instructions by trained technicians and were conducted at a central laboratory (Vespasiano, MG, Brazil).

### 2.3 Statistical analysis

All analyses were performed using SPSS Statistics, version 22.0 (IBM Corp, Armonk, NY). The analyses incorporated data weighting, clustering (as selection commissions with different sizes were included), and stratification. Since the data set was obtained using a complex sampling procedure that combined stratification and clustering, the design of the survey was incorporated into the statistical analysis of the data. Additionally, a calibration procedure had to be applied for the samples according to the census distribution by population size of the city of residence (<80,000 inhabitants, 80,000–199,999 inhabitants, and ≥ 200,000 inhabitants), as well as educational levels.

Qualitative variables were presented as absolute and relative frequencies, and quantitative variables were presented as means and standard deviations (SDs). The syphilis prevalence was expressed with a binomial confidence interval (CI) of 95%.

In the multivariate analysis, logistic regression analysis was used to investigate the factors that were mostly associated with syphilis infection in 2016. Initially, univariate logistic regression analysis was used to calculate crude odds ratios. In the multivariate analysis, all variables potentially associated with syphilis infection were included as follows: educational levels; MSM status; more than 5 casual partners within the last year; more than 10 partners throughout ones’ lifespan to date; consistent condom use; paid or received money for sex; a reported history of STIs in ones’ lifetime; having the 1st sexual intercourse encounter before 15 years of age. A stepwise procedure was used for the selection of joint variables associated with syphilis, with variables included and excluded in each step based on the likelihood ratio test. *P*-values <.05 were considered statistically significant.

### 2.4 Ethics

This study was approved by the Brazilian National Commission of Ethics in Research (CONEP), register number 278.616 on May 21, 2013. This study also obtained approval from the local Institutional Review Board of the coordinating center (Universidade de Caxias do Sul, Caxias do Sul, RS, Brazil; register number 1.074.338) on May 22, 2015; this was updated on February 24, 2016 (register number 1.422.093). All participants signed a written consent form.

## 3 Results

### 3.1 General characteristics

In total, 38,247 conscripts aged 17 to 22 years were enrolled in the study; 965 (2.5%) were excluded due to lack of information regarding age, origin (municipality), and educational levels. Thus, 37,282 (93.2%) conscripts across Brazil were included in the study. The general characteristics of the study population have previously been described in detail.^[14]^ Briefly, the mean age of the participants was 18 years (SD: 0.8), 98.2% (n = 36,436) were single, and 93.6% (n = 34,894) lived with their parents or relatives. Regarding the educational levels, 93.5% (n = 34,860) of conscripts completed elementary education, 50.7% (n = 18,908) completed high school, and 67.0% (n = 24,852) were still in school. Most participants reported that their mothers had completed an elementary (55.8%, n = 20,720) or high (41.2%, n = 15,306) school education, whereas educational levels reported for their fathers were 47.2% (n = 17,415) and 33.9% (n = 12,514) for completed elementary and completed high school, respectively.

Generally, the distribution of the conscripts across regions and urban/city levels resembled that of the Brazilian population.^[15]^ The Southeast and Northeast regions contributed with the largest number of conscripts (39.2% and 29.4%, respectively), followed by the South (13.9%), North (9.9%), and central-west (7.6%) regions.

### 3.2 Syphilis prevalence

The estimated syphilis prevalence rates across the country by region are shown in Table 1. The estimated syphilis prevalence rates were: 1.63% (n = 584) for screened and 1.09% (n = 393) for confirmed syphilis, ranging from 0.87% in the central-west region to 2.27% in the North region and from 0.68% in the central-west region to 1.48% in the South region, respectively.

**Table 1:**
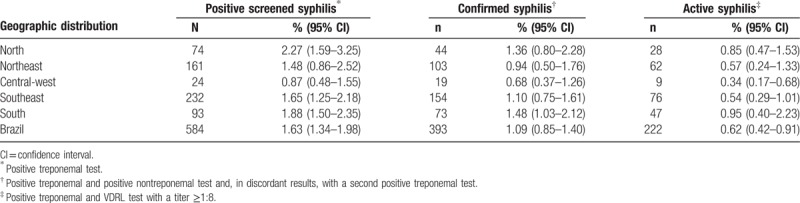
Estimated prevalence of screened, confirmed, and active syphilis among conscripts by region in Brazil, 2016.

Among participants who screened positive for syphilis, the proportions of participants who tested positive for HIV and hepatitis B infection were 1.4% and 0.1%, respectively, whereas among confirmed syphilis conscripts the proportion of individuals who tested positive for HIV was 1.2% and no hepatitis B co-infected participants were identified. No participants were found to be co-infected with syphilis and hepatitis C (data not shown).

The estimated syphilis prevalence rates for the MSM population were 5.23%, 4.61%, and 3.60% for screened, confirmed, and active cases, respectively.

The estimated active syphilis prevalence rates across the country by region are shown in Table 1. The South region had the highest active syphilis prevalence rates (0.95%, n = 47), whereas the active syphilis infection prevalence rate for the entire country was 0.62% (n = 222).

### 3.3 Risk factors

A total of 25,752 (67.3%) conscripts had already had sexual intercourse involving penetration and were therefore considered to be sexually active. Among the screened and confirmed syphilis positive conscripts, approximately 14% reported that they had never had sexual intercourse. Table 2 summarizes the risk factors associated with positive screened syphilis and confirmed syphilis among the sexually active conscripts (73.7%), when compared with the syphilis negative conscripts. The sexual behaviors that were more frequently associated with confirmed syphilis conscripts were: sexual relationships with casual partners in the last year (66.0% vs 40.2%, *P* < .001), same-sex sexual relationships to date (MSM) (16.3% vs 4.3%, *P* < .001), more than 10 partners (39.6% vs 20.2%, *P* = .006), and sexual intercourse before 15 years of age (54.8% vs 31.9%, *P* = .003).

**Table 2:**
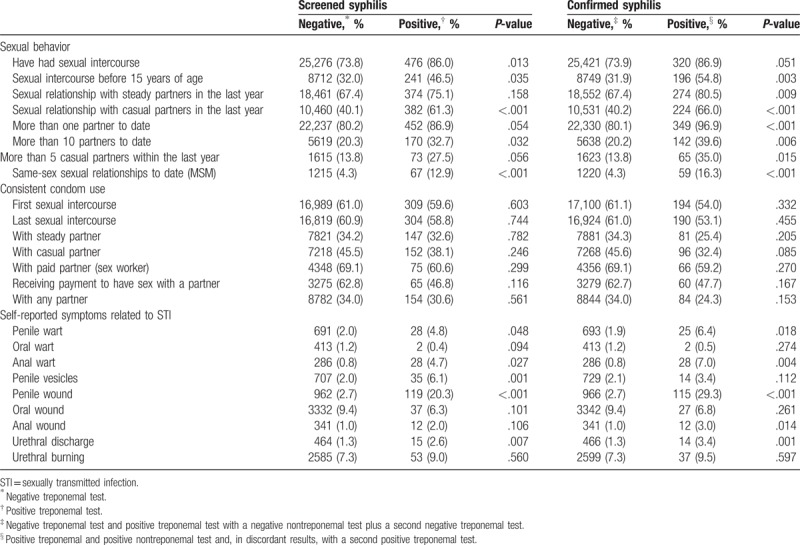
Risk factors associated with a positive screening test and confirmed syphilis diagnosis in Brazilian conscripts, 2016.

Although not statistically significant, only 25.4% of the conscripts who had confirmed syphilis reported the use of condoms with steady partners, 32.4% with casual partners, and only 24.3% with any partner. Among the MSM population, 78.1% reported inconsistent use of condoms compared to 65.4% of their non-MSM counterparts (*P* = .016).

The STI symptoms analyzed in this study that were more frequently reported by conscripts with confirmed syphilis included: penile wounds (29.3% vs 2.7%, *P* < .001), urethral discharge (3.4% vs 1.3%, *P* = .001), anal warts (7.0% vs 0.8%, *P* = .004), and penile warts (6.4% vs 1.9%, *P* = .002).

Table 3 indicates some of the risk factors associated with the geographic region. The most common self-reported STI symptom was oral wounds (9.3%), followed by urethral burning (7.2%) and penile wounds (2.9%). We found evidence of an association between the Northeast region and self-reported penile warts (2.8%) (*P* = .002). Of note, 43.9% of the participants who had any symptoms did not seek care or the problem disappeared without treatment; another 26.7% and 21.1% sought care at public health services and from family members, respectively. The most commonly self-reported STIs were gonorrhea (1.5%), herpes (1.2%), human papilloma virus (0.7%), syphilis (0.6%), and HIV (0.5%).

**Table 3:**
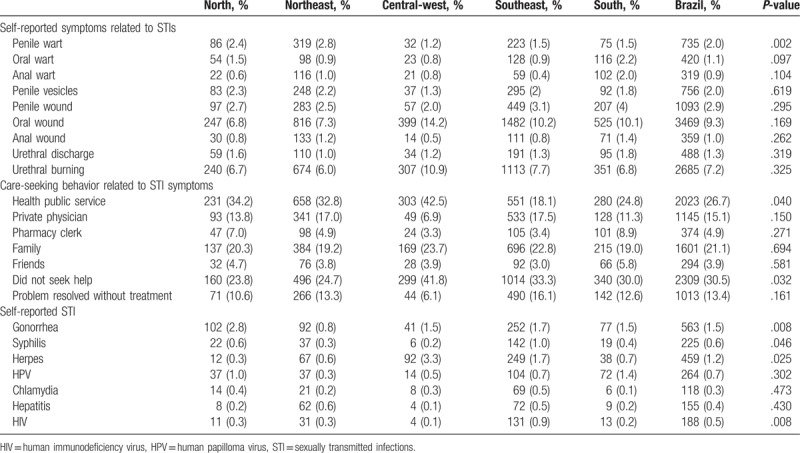
Self-reported sexually transmitted infections and symptoms related to sexually transmitted infections by geographic region in Brazil, 2016.

Additionally, regarding serologic status, we found that among those with active syphilis, 81% reported not having had syphilis infection in their lifetime.

The findings of the multivariate logistic regression analysis with the syphilis test as the response variable are presented in Table 4. The independent factors associated with syphilis infection were: a self-reported history of having STIs in ones’ lifetime (OR = 7.24; 95% CI = 3.99–13.13; *P* < .001), MSM status (OR = 3.43; 95% CI = 1.73–6.79; *P* = .001), and having their 1st sexual intercourse encounter before 15 years of age (OR = 2.62; 95% CI = 1.36–5.05; *P* = .004).

**Table 4:**
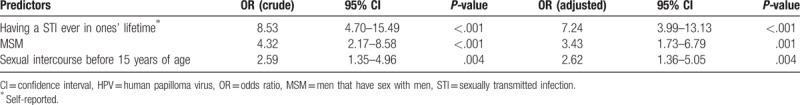
Multivariate analysis of the independent risk factors for confirmed syphilis infection among conscripts in Brazil, 2016.

No association was found between syphilis infection and other variables, such as educational level, more than 10 partners, more than 5 casual partners within the last year, or consistent condom use among those who paid or received money for sex (*P* > .05).

## 4 Discussion

Syphilis rates and trends vary according to population subgroups; in this context, estimating the prevalence of infection and identifying key risk factors for syphilis acquisition allows us to better target prevention strategies. This study provides a comprehensive approach to the syphilis problem among the young male population in Brazil, and it was conducted as part of a series of surveys with Brazilian army male conscripts to assess the syphilis prevalence and associated risk factors. As expected, due to the age range (17–22 years) of the study population, most of the young men were single, lived with their parents, and had at least an elementary education.

We found an increase in the estimated prevalence of positive screened syphilis among Brazilian army male conscripts from 0.55% in 2007 to 1.63% in 2016.^[13]^ The South region had the highest increase in the estimated syphilis prevalence (from 0.26% to 1.88%) followed by the North region (from 0.85% to 2.27%), Southeast region (from 0.34% to 1.65%), Northeast region (from 0.82% to 1.48%), and the Central-west region (from 0.49% to 0.87%). Our data are in agreement with the 2017 Brazilian Syphilis Epidemiological Bulletin, which reported an increase in the number of syphilis notifications in patients aged 13 to 29 years from 2010 to 2016.^[4]^ To our knowledge, this is the 1st study to report the estimated prevalence of confirmed syphilis (1.09%) and active syphilis (0.62%) among the young male population in Brazil.

A lack of awareness regarding serostatus was observed in the study population; 81% of the young males who were diagnosed with active syphilis actually reported not having the infection. Testing and diagnosis are important 1st steps in receiving treatment and reducing transmission. Although the Brazilian Ministry of health promotes regular testing campaigns, it seems that testing efforts are not adequately reaching this group.

As shown in a previous survey among Brazilian conscripts, our study showed an association between HIV infection and STI-related symptoms; however, in our study gonorrhea was the most commonly self-reported STI.^[13]^ Syphilis, gonorrhea, and chlamydia may not present with any symptoms or have unspecific clinical manifestations, which may have been mistakenly self-reported as similar symptoms by conscripts.

Self-reported STI symptoms were more often reported by syphilis positive conscripts; penile wounds were the most frequently reported symptoms. Of note, almost half of the conscripts did not seek health care assistance or the symptom disappeared without treatment. Men's health care seeking behaviors usually make them less willing to overcome the practical barriers they encounter, including the lack of extended opening hours at clinics, the inconveniently located facilities, difficult-to-use booking systems, long delays between making an appointment and seeing a clinician, and unpredictable waiting times on the day of the appointment.^[16]^

It is important to highlight that sexual transmission of syphilis occurs during primary, secondary, and early latent stages of infection. The initial disease course presents with symptoms that are typically painless and that resolve spontaneously; this may also explain why individuals do not seek health services in a timely manner.^[2,17]^ Thus, those with no signs of infection serve as reservoirs for the ongoing spread of infection because the absence of symptoms does not preclude transmissibility.^[2,18]^

Currently, the high rates of syphilis infection seem to be disproportionately observed in men, specifically on MSM.^[17–22]^ Data obtained in this study showed a prevalence of 5.23% among conscripts who had same-sex relationships. We found that young men who had already had an STI in their lifetime were approximately 6 times more likely to have syphilis, and although the proportion of conscripts who were MSM was 4.3%, MSM were 2.43 times more likely to have syphilis than their non-MSM counterparts. Recent studies conducted in different regions of Brazil showed syphilis prevalence among MSM ranging from 9% to 26.3% emphasizing the need for interventions among this group.^[6–9,13,19,23]^

Variables associated with syphilis in this study were: sexual intercourse before the age of 15 years, having history of STIs ones’ lifetime, and same-sex sexual relationships (MSM). Similarly, as shown by Rocha et al (2018) there was a significant association between sexual intercourse before the age of 15 years and an increased sexual risk behavior among the MSM evaluated. These findings indicate that the earlier the onset of sexual activity, the more frequently participants partook in risk behaviors for syphilis infection.^[19]^ This increased risk could be explained by the fact that youth have difficulties in negotiating condom use with partners in the beginning of their sexual life. Thus, the urgency of having new experiences might lead them to higher risk sexual behaviors and the spontaneity or unplanned sexual intercourse encounters are generally associated with the lack of condom use. Additionally, as was previously found by Ribeiro et al (2012), young adults presenting with other STIs, who had their 1st sexual encounter at a young age or who were MSM, were at greater risk of syphilis infection.^[13]^

As there is no vaccine against syphilis, the most effective prevention method continues to be the use of condoms, male circumcision, and the avoidance of sex with infected partners. Additionally, there should be prompt treatment to avoid continued sexual transmission or mother to child transmission, and the treatment of all sexual partners to avoid reinfection.^[17]^ Our results, however, indicate that condom use was infrequent, especially among MSM who had the lowest frequency of condom use.

Additionally, our results are similar to those of the previous surveys on young conscripts of the Brazilian Army that indicated that the regular use of condoms with steady and casual partners was approximately 40% and 50%, respectively. The lowest frequency of condom use was among young MSM.^[12]^

Our findings are similar to those from a study carried out in Brazil that compared descriptive sociobehavior characteristics in 2 national cross-sectional HIV biologic behavioral surveillance surveys conducted among MSM. The findings from this previous study showed that from 2009 to 2016, there was an increase in unprotected anal receptive and insertive sex, with very high rates in 2016.^[24]^

There is a downward trend in the consistent use of condoms for many reasons. These include HIV no longer considered a “death sentence,” wider antiretroviral-based biomedical interventions for HIV prevention (pre- and postexposure prophylaxis), increased risk-taking behaviors, the reduction in intervention programs and changes in the way in which individuals find partners (social networks).^[17,25]^

This study was subject to several limitations. First, illiterate conscripts were dismissed from military service and were not enrolled in the study. Second, self-reported questionnaires were used, which may have led to losses due to inadequate responses. Third, syphilis diagnosis did not include a clinical assessment. Finally, the criteria used for the definition of active syphilis (a VDRL titer of 8 or greater and a treponemal-positive test) may include cases of untreated latent syphilis or cases of individuals successfully treated in whom the VDRL could remain positive for a long period of time. Nevertheless, these limitations are minimized by the cross-sectional study design, and the use of standardized data collection instruments and laboratory measurements, as well as the large sample size.

Syphilis remains as an important public health issue and understanding the dynamics of this disease and its prevalence in subgroups is necessary to help policymakers devise targeted prevention strategies and improve the quality of care offered to those affected. Effective interventions are needed to achieve the goal of a 90% reduction in the incidence of syphilis globally by 2030 as set by the WHO.^[26]^

## 5 Conclusion

Our data suggest that syphilis is on the rise across the Brazilian young male population. The estimated syphilis prevalence rates for the entire country were: 1.63%, 1.09%, and 0.62% for screened, confirmed, and active syphilis, respectively. This increasing prevalence, particularly among MSM, indicates the need for urgent public health interventions, action plans, and the implementation of risk reduction strategies focused on this population. Further research is recommended to assess the syphilis seroprevalence among the illiterate male population and to better understand the acquisition dynamics of the syphilis infection and social and sexual networking specifically in the MSM population.

## Acknowledgment

The authors acknowledge the Brazilian Ministry of Defense and all participants. They also thank Dr Célia Landmann Szwarcwald and her team at FioCruz, Rio de Janeiro, RJ, Brazil for their statistical assistance.

Leonardo Rapone da Motta orcid: 0000-0003-3673-2687.
